# Bioprinting for Liver Transplantation

**DOI:** 10.3390/bioengineering6040095

**Published:** 2019-10-10

**Authors:** Christina Kryou, Valentina Leva, Marianneza Chatzipetrou, Ioanna Zergioti

**Affiliations:** Department of Physics, National Technical University of Athens, 15780 Zografou, Greece; chkryou@central.ntua.gr (C.K.); vleva@mail.ntua.gr (V.L.); mchatzip@mail.ntua.gr (M.C.)

**Keywords:** additive manufacturing, direct printing, 3D structuring, tissue engineering

## Abstract

Bioprinting techniques can be used for the in vitro fabrication of functional complex bio-structures. Thus, extensive research is being carried on the use of various techniques for the development of 3D cellular structures. This article focuses on direct writing techniques commonly used for the fabrication of cell structures. Three different types of bioprinting techniques are depicted: Laser-based bioprinting, ink-jet bioprinting and extrusion bioprinting. Further on, a special reference is made to the use of the bioprinting techniques for the fabrication of 2D and 3D liver model structures and liver on chip platforms. The field of liver tissue engineering has been rapidly developed, and a wide range of materials can be used for building novel functional liver structures. The focus on liver is due to its importance as one of the most critical organs on which to test new pharmaceuticals, as it is involved in many metabolic and detoxification processes, and the toxicity of the liver is often the cause of drug rejection.

## 1. Introduction

Over the past few decades, printing technology has advanced from two-dimensional (2D) printing to an additive process in which successive layers of material are arranged to create 3D objects [1,2]. The ability of printing techniques to produce 3D structures with complex geometries and structures enables rapid prototyping and manufacturing in the industry, as well as the production of personalized medicine.

The 3D printing field was first introduced in 1986 by Charles W. Hull as “stereolithography” [3]. In this technique, thin layers of a material were printed in layers to form solid 3D structures using photochemical processes. Since the 1990s, stereolithographic models have been used for creating sacrificial resin molds for the formation of 3D scaffolds of biological materials. Those materials are used for transplantation with or without seeded cells [4]. The next generation was “3D bioprinting,” which was used as a tool for tissue engineering and organ fabrication.

3D bioprinting employs the controlled, precise delivery and placement of living cells, biomaterials and biochemicals to fabricate functional 3D constructs in a layer by layer manner. 3D bio-printing has emerged as one of the most influential applications of 3D printing, aiming to address the increased demand for living constructs with long term mechanical and biological stability, suitable for transplantation and improved drug discovery models [5,6]. 3D bio-printing permits rapid manufacturing with high-precision and control over size, as well as adjustments to the shape, porosity, and mechanical strength of the scaffolds in one step; it has thus attracted much attention in the tissue engineering field. One of the main drawbacks of the 3D bioprinting technologies is the vascularization of the created tissue structure, which still remains a critical challenge. The development of vascular networks within densely populated and metabolically functional tissues facilitate the transport of nutrients and oxygen, and it provides a way to remove wastes, for which the long term preservation of cellular viability can be obtained. Moreover, it has been considered as a promising method to replace defective or damaged tissues or organs in which scaffolds have functioned as carriers for cell interaction and provided physical support to the freshly developed tissue [7].

Impressive progress has been accomplished in fabricating complex tissue constructs in the past few years. The main approaches for controlled 3D vascularization within these engineered tissues mainly involve microfluidic-based technologies [8,9]. The microfluidic-based technologies can provide a versatile platform for engineered tissues because they can create complex and functional micro-scale environments in order to mimic 3D in vivo environments (e.g., a chemical gradient). Moreover, microfluidic technologies have emerged as useful tools for complex cell environments like tissues due to the integration of multiple steps and fluid control, such as controllable cell culture, cell capture, mixing, genetic assays, protein and continuous nutrition, and oxygen supply [10,11,12,13]; however, they are also limited by fabrication complexity. A functional circulatory system is a key factor for the creation of tissue constructs which are limited to a distance of just a few hundred microns but are not limited to diffusion for nutrition [14]. In addition, innovative strategies such as the guided infiltration of host micro vessels into the implanted construct, the integration of autologous vascular grafts, and the direct bioprinting of vascular structures have also been attempted by the research community [15].

This review aims to highlight the techniques used for the patterning of cells towards the creation of a structures with increased complexity such as tissues and organs. Special attention is given to the techniques used for the fabrication of tissue structures such as the creation of 3D scaffolds and/or direct printing techniques, as well as the combination of both approaches.

## 2. 3D Bioprinting Techniques

The liver is an extremely important organ for functions related to metabolism and metabolic regulation. Unfortunately, liver failure or acute chronic liver failure remains one of the most major causes of mortality in the world. As a result of the increase in liver diseases, the need for donor organs is increasing [16]. Despite the great importance of the organ in a human’s life, liver transplantation is usually performed only on patients with major and/or end-stage liver diseases due to the short life span of donor organs or rejection risk. Consequently, alternative methods, including tissue engineering, are needed and are actively being pursued. The field of liver tissue engineering includes several techniques aimed at providing therapeutic development for liver diseases and plays an important role in the mechanistic understanding of liver biology interactions in healthy and diseased states in a high throughput platform. Artificial liver transplantation is a recent challenge in medicine, as it has been deemed the best therapeutic method for severe liver diseases. Conventional liver tissue models have recently been used to fabricate in vitro 3D liver tissue models [17]. These methods can be classified into four main categories: (i) Monolayer cell cultures, including aggregating and assembly techniques; (ii) hollow fiber; (iii) suspension chambers; and (iv) perfusion beds [18]. Nevertheless, these approaches often fail to imitate the complexity of native liver tissue and are incapable of depositing multiple cell types in desired patterning [19].

Three-dimensional (3D) printing, which belongs to the family of additive manufacturing techniques [20], can resolve issues inherent to traditional 2D and 3D models, such as the low efficacy of engraftment and poor cellular functions, because 3D printing provides the ability to manipulate cell–cell interactions as opposed to conventional models.

3D printing was first developed in the 1980s, and there have been enormous advancements in tissue and organ regeneration [21]. Ιn 1993, the first 3D printer was designed by Sachs et al. to print nonviable materials, such as plastics and metals [22]. Since then, a number of 3D printers [23] have been successfully designed and used for tissue biofabrication and regenerative medicine [5,24] Typically, 3D bioprinting starts with a computer-aided process for depositing biological materials such as living cells, matrices, biomaterials, and molecules in a layer by layer manner with a prescribed configuration in order to produce scalable bioengineered structures [25]. In this way, 3D biomimetic tissue models with heterogeneous cell placements and vasculature have been proposed as means to recapitulate liver tissue complexity and architecture [26]. The fabrication of perfectly functional liver networks remains a challenge for most tissue engineers. Hence, there are considerable types of 3D printing methods that are expected to overcome current limitations. 3D bioprinting offers the ability to develop highly complex 3D patterns with living cells that mimic organ level functions, and it has therefore been applied in organs-on-chips and organs engineering. The main bioprinting techniques are extrusion [27], inkjet [28] and laser-induced forward transfer (LIFT) [29,30], each one possessing several advantages and disadvantages.

### 2.1. Laser Bioprinting

**Figure 1:**
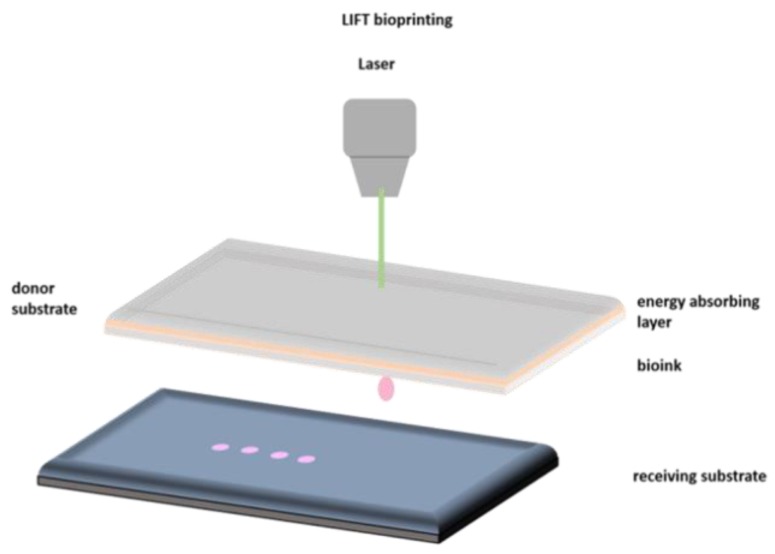
Schematic representation of laser-induced forward transfer (LIFT) setup.

Laser-induced forward transfer (LIFT) is a technique presented more than 30 years ago by Bohandy et al. [31]. Briefly, a pulsed laser beam is applied on a donor slide (or ribbon) covered with a laser-energy-absorbing layer (e.g., gold or titanium) containing the desired material (e.g., cells, hydrogels and growth factors), followed by the evaporation of the material; this results in a high-pressure bubble jetting toward the receiving substrate that is placed underneath the donor slide, as shown in Figure 1.

For the direct printing of cells, the use of LIFT is proposed because it enables the printing of bio-inks within a wide range of viscosities (1–300 mPa s) [32] and at high speeds while cell viability is preserved (>90%).

LIFT is a nozzle-free technique and therefore does not have the problems of nozzle clogging with cells or biological materials, which are some major drawbacks of other bioprinting technologies. Moreover, this technique offers printing cell concentrations up to 1 × 108 cells/mL with a very high resolution [33].

The use of LIFT for the printing of functional biomaterials can be traced back to 2003 [34], while the development of 2D cell structures was first proposed in 2008 [35]. Regarding the use of lasers for the printing of 3D structures, the first report was published in 2011 by M. Gruene et al. [36], while in 2012, Koch et al. [37] published the printing of multiple cell lines in order to create epidermal tissue. These multiple cell lines were previously proven to be resistant to damage during the laser-assisted printing process [38]. The proliferation of cells over a period of 10 days was studied, and the ability of 3D printed cells to form real tissue was demonstrated. It is critical to know how the laser process affects cell viability as well as phenotypes. Catros et al. [39] studied the effects of laser pulse energy, extracellular matrix (ECM) thickness and viscosity of the bioink on cell viability. Cell viability 24 h post-printing was measured to compare different printing settings. It was concluded that while higher laser energy leads to more cell fatality, increasing film thickness as well as bioink viscosity results in increased cell viability. Moreover, another laser group investigated the effects of bioink viscosity, laser energy and printing speed on printing resolution [32]. It was shown that a microscale resolution and 5 kHz printing speed were within reach. This work is another proof for the applicability of printing cells and biomaterials via LIFT printing to engineer miniaturized tissue layouts with de novo high cell density and microscale organization. An interesting study was demonstrated by Keriquel et al. [40], whereby in vivo laser bioprinting was used to deposit nano-hydroxyapatite in a mouse calvaria 3D defect model as a proof of concept. In the future, study materials that can directly integrate into a patient’s tissue could be used. Finally, incorporating the patients’ own cells may facilitate the applicability of these types of constructs to contribute to both the structural and functional components of the tissue.

### 2.2. Inkjet Bioprinting

Inkjet-based bioprinting is a noncontact technique in which droplets of cells or biomaterials are patterned into desired substrates.

**Figure 2:**
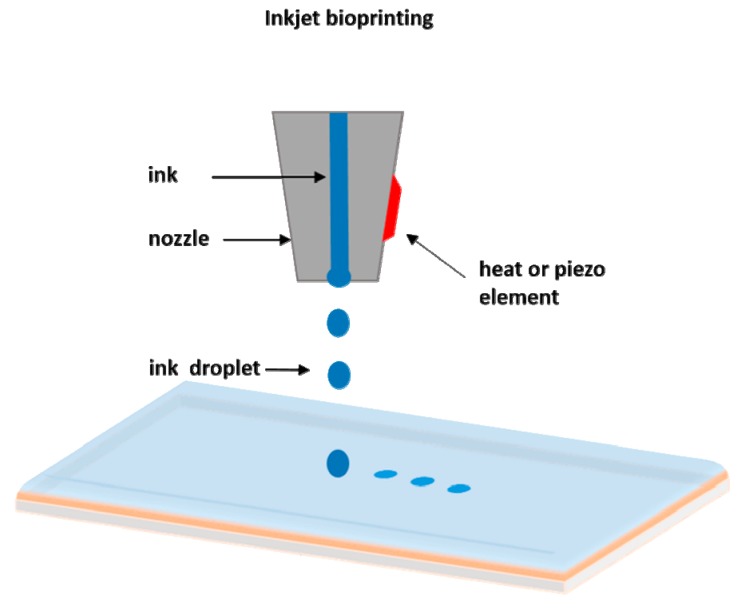
Schematic representation of inkjet printing.

The drop-on demand inkjet bioprinters are the most common ones, and they consist of thermal, piezoelectric, and electrostatic inkjet nozzles [41]. A schematic diagram of inkjet printing is shown in Figure 2.

With respect to the construction of cellular structures, inkjet bioprinters are normally used for the printing of matrices for the cell growth, such as small scaffolds. Different inkjet printheads with multiple nozzles have been developed to increase printing speed and fabricate larger cellular constructs [42].

However, inkjet bioprinters also have limitations on material viscosity (ideally below 10 centipoise) due to the excessive force required to eject drops using solutions at higher viscosities [43]. Another major disadvantage of this technique is the difficulty in achieving biologically relevant cell densities. Often, low cell concentrations are used to facilitate droplet formation (less than 10 million cells/mL) [44]. To provide a higher concentration of cells, the inhibition of some hydrogels can be generated by adding crosslinking agents. However, the requirement for crosslinking agents often slows the bioprinting process and involves the chemical modification of naturally occurring ECM materials, which changes both their chemical and material properties [45]. Despite these disadvantages, inkjet bioprinting has notable benefits, including low cost, high speed and biocompatibility with a broad range of biological materials [46]. Significant studies of inkjet printing have included the regeneration of functional tissues, such as skin and cartilage, in situ [47,48]. With the advantages of high throughput digital control and high resolution, this technique enables the direct placement of cells, biological factors and biomaterial scaffolds directly into skin or cartilage lesions. Inkjet-based bioprinting facilitates the successful deposition of either primary cells or stem cell types with uniform density, and it maintains high cell viability and function after printing. These studies have shown the ability of inkjet bioprinting to regenerate functional constructs.

### 2.3. Extrusion Bioprinting

**Figure 3:**
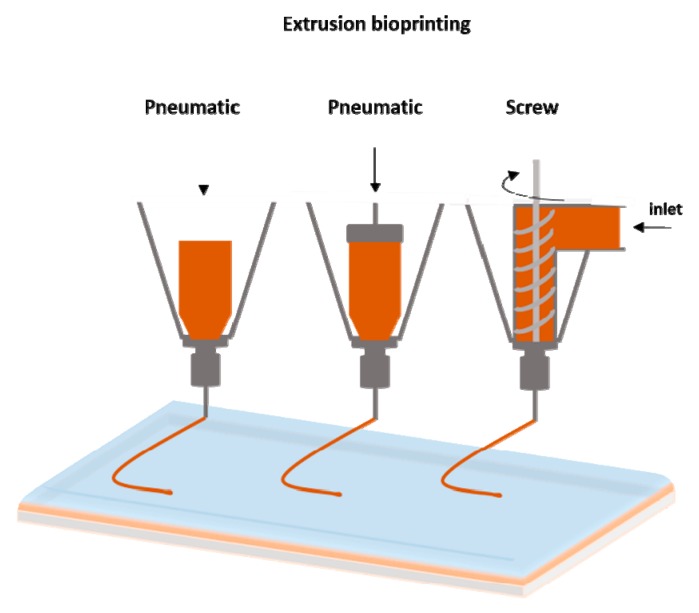
Schematic representation of extrusion bioprinting.

The extrusion-based bioprinting technique is characterized by a temperature-controlled biomaterial dispensing system driven by a pneumatic pressure or a mechanical piston, as demonstrated in Figure 3. Schematic representation of extrusion bioprinting. The printing system generates continuous biomaterial filaments, instead of droplets, that are deposited in two dimensions; filaments are placed along the *x*- and *z*-axes and then move higher in the y-axis. The final product is a 3D structure. This technique provides the ability to deposit very high cell densities as well biological material such as hydrogels and biocompatible copolymers. Several groups have used sole cells or multicellular cell spheroids and allowed for their self-assembly into the desired 3D structures using extrusion bioprinters [49,50,51]. Pioneer work using this approach is currently being performed at the Wyss Institute under Prof J. Lewis [52]. Each print head is equipped with an on-board temperature controller to adjust the temperature depending on the material that is being printed, enabling sequential layer-by-layer printing and avoiding contamination between different materials.

However, a major disadvantage of extrusion bioprinting is that cell viability is lower than that with inkjet-based bioprinting (40–86%). The decreased cell survival rate possibly results from the shear stresses inflicted on cells in viscous fluids [53].

Extrusion-based bioprinting approaches have been also used for the generation of multiple tissue types, including aortic valves [54] and in vitro pharmokinetic models [55].

A review of the outstanding research works using the above printing techniques for liver and liver tissue engineering is presented.

A brief review of the above mentioned bioprinting techniques is presented in Table 1. A brief review of common bioprinting techniques.

## 3. Tissue and Liver Bioprinting

As previously mentioned, the liver is considered one of the most significant organs in the human body due to its special characteristics. It plays a major role in metabolism with numerous functions, including the regulation of glycogen storage, the decomposition of red blood cells, plasma protein synthesis, hormone production, and the detoxification of chemicals [56,57]. In anatomy, the liver is divided into four lobes. The right lobe, which is much bigger than the left lobe, involves two minor lobes—the quadrate and caudate lobes. Blood is supplied to the liver through two different vessels. The hepatic artery supplies arterial blood from the heart to the liver, and the hepatic portal vein carries blood consisting of nutrients and toxins from the intestines to the liver [57].

**Figure 4:**
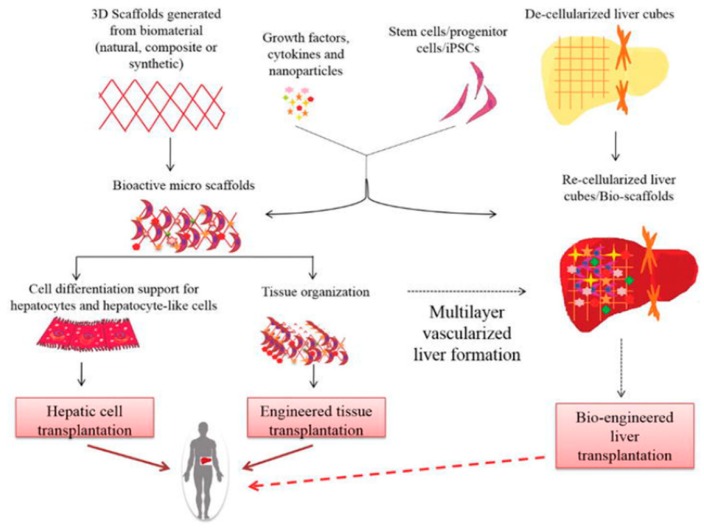
Schematic diagram of liver tissue engineering. Solid lines show already ongoing approaches, whereas dotted lines indicate proposed mechanisms [59].

The liver has an extensive regeneration capacity due to the high proliferation ability of hepatocytes, even if it is subjected to vast damages. The tissue engineering of the liver is not new, and there are several groups that have worked on the engineering of liver tissues and bioartificial livers as early as 1996 [58,59]. Therefore, various tissue bioprinting techniques have been used to fabricate biomimetic liver tissues—even a whole liver. A schematic representation of the key approaches used for liver tissue engineering is demonstrated in Figure 4 [59].

### 3.1. Micropatterned 2D and 3D Liver Models

Over the past few decades, liver tissue engineering has made significant progress towards the establishment of in vitro liver models for both fundamental pathophysiological studies and drug screening. The sources of cells used for these in vitro liver models include primary hepatocytes, hepatic cell lines isolated from tumors or liver slices, and stem cell-derived hepatic cells [60,61]. Griffith et al. [62] fabricated a vascularized liver on a small scale using the inkjet printing technique. They were pioneers in investigating the role of scaffold architecture from biodegradable polyesters using a manufacturing technique amenable to scaling-up, commercial production, and culture conditions for achieving hepatic function in long-term perfusion cultures.

Monolayer culture, organoid culture and co-culture platforms have been established using culture plates [63], commercially available wells [64], dielectrophoresis micropatterning [65] and physical mask-based additive photopatterning methods [60]. However, the liver specific functions of hepatocytes cultured in such platforms are functional only for weeks of in vitro culture [63,66]. Therefore, liver constructs that better mimic the native environment and help maintain in vitro liver functions is in great demand.

3D bioprinting technology, with its potential to pattern cells and biomaterials in a precise manner, provides a great tool to achieve novel and biomimetic in vitro liver models with increasing structural complexity.

### 3.2. 3D Bioprinting for Liver Models

3D printing is a scientific field with innovative techniques that offer remarkable benefits in terms of the vascular network formation of liver tissues and organs due to their feasibility, variety of available printing methods, and precise controllability. With the appearance of bioprinting, the constructions of functional tissue livers or mini liver organs have become an impending reality. Currently, many researchers are contributing to the improvement of 3D printed vascular networks on a best effort basis for their introduction into the medical field.

**Figure 5:**
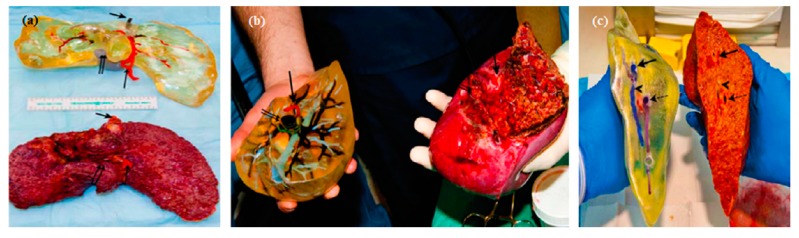
(**a**) Side view of a 3D printed liver and extracted liver of a patient, where long, short, and double arrows indicate hepatic artery, hepatic vein, and portal vein, respectively. (**b**) Right lobes of 3D printed and extracted livers with indications of the hepatic artery (single arrows) and portal vein (double arrows). (**c**) Cross-sectional views of 3D printed and extracted livers with indications of hepatic vein (single arrows) and portal vein (dotted arrows) [70].

Many researchers that have worked on tissue engineering have successfully achieved to fabricate biomimetic 3D printed vascularized liver constructs with their own unique properties such as rapid restoration ability even after considerable damage [67]. In an earlier work by Cheng et al. [68], 30 layers of a hepatocyte/gelatin mixture were laminated into a high spatial structure using a 3D rapid prototyping technology. The 3D hepatocyte/gelatin pattern remained viable and performed biological functions in the construct for more than two months. In an effort to develop personalized tissues and organs for precision medicine, Organovo, harnessing the advantages of 3D bioprinting, used a syringe-based extrusion printer to develop 3D printed human liver tissues that can remain fully functional and stable for up to 28 days. The researcher demonstrated a multicellular liver structure involving hepatocytes, hepatic stellates, and endothelial cells (ECs). 3D liver tissues possessed critical liver functions, including albumin production, cholesterol biosynthesis, fibrinogen and transferrin production, and inducible cytochrome (CYP) 1A2 and CYP 3A4 activities. These in vitro models of 3D vascularized livers could potentially be implanted into patients to replace their damaged livers [69]. In 2013, the first human liver was synthetically reproduced and validated against the actual native liver at the time of surgery by Zein et al. [70]. Specifically, successful 3D synthetic livers were printed along with their complex network of vascular and biliary structures which replicated the native livers for six patients, three living donors, and three respective recipients. Prior to the transplantation, the dimensions of the donor and recipient livers were recorded in detail, including the diameters of veins to fabricate a vascularized liver using the inkjet printing technique and based on each patient’s individual computed tomography (CT) scan and magnetic resonance imaging (MRI). To implement external vascularization, the authors utilized a permanent adhesive to attach to the liver lobe (Figure 5). These results demonstrate the potential efficacy of a 3D printed synthetic liver with a vascular network in the human body as a valuable tool for drug delivery, a substitute for treating partially or irreversibly damaged liver tissue, and a tool for potentially minimizing intraoperative complications. That was the first human liver to have been synthetically reproduced and validated against the actual native liver at the time of surgery.

**Figure 6:**
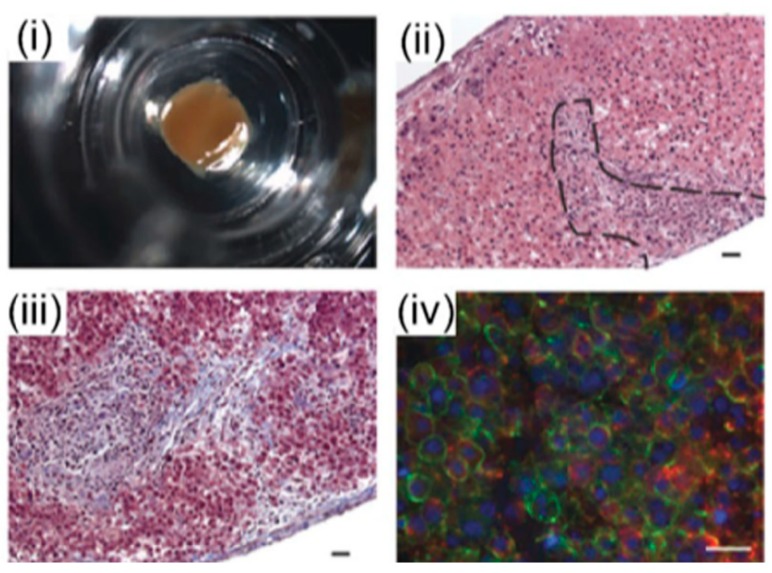
Organovo’s mini liver tissue: (**i**) A macroscopic image of liver tissue housed in a 24-well transwell, (**ii**) Hematoxylin and eosin (HE) staining of a tissue cross-section, (**iii**) extracellular matrix (ECM) deposition assessed by Masson’s trichrome staining, and (**iv**) Ιimmunohistochemistry (IHC) staining of the parenchymal compartment for E-cadherin (green) and albumin (red) [71].

Nguyen et al. [71], established a novel bioprinted human mini liver tissue from the co-culture of primary human hepatocytes, hepatic stellate cells (HSC) and human umbilical vein endothelial cells (HUVEC) cells to test clinical drug-induced toxicity in vitro using an inkjet 3D bioprinter. A histological analysis showed the presence of distinct intercellular hepatocyte junctions, cluster of differentiation 31 (CD31+) endothelial networks, and desmin-positive, smooth muscle actin-negative quiescent stellates, mimicking the in vivo human drug response at the tissue level (Figure 6). A major challenge in liver tissue engineering is the proliferation, long-term culture and maintenance of hepatocyte function ex vivo of primary hepatocytes [38].

A recent study by our team [72] utilized the LIFT technique to laser print hepatocyte cancer cell line Huh7 on porous collagen-Glycosaminoglycan (GAG) scaffolds, which are biomaterials with established applications in re-generative medicine implants. The results showed the benefits of the laser bioprinting technique for the precise placement and immobilization of hepatocyte cells into porous collagen scaffolds for novel custom-made implants for regenerative medicine applications.

Arai et al. [73] used an inkjet 3D bioprinter to fabricate a 3D culture system using an artificial scaffold for studying the liver-specific functions of hepatocytes. The printed construct expressed liver-specific proteins and receptors such as albumin, MPR2, and asialoglycoprotein receptor (ASGPR), thus proving the functionality of the printed liver tissue. The work by Matsusaki et al. [74] demonstrated that high cell activities and high cell–cell interactions of the fabricated 3D human liver chip from HepG2/HUVECs laden fibronectin and gelatin using inkjet printing technology were analogous to the native liver structure due to the hierarchical sandwich structures.

In another study by Y Kim et al. [75], mouse primary hepatocytes (isolated from the livers of six-to-eight weeks old mice) were printed into a 3D liver tissue construct using an extrusion-based bioprinting system. Cells were viable for 14 days, with liver-specific gene expressions, namely albumin, hepatocyte nuclear factor 4 alpha (HNF-4α), forkhead box protein A3 (Foxa3), and asialoglycoprotein receptor 1 (ASGR1), increasing gradually up to day 14. In another study, Lee et al. [76] developed 3D structures from polycaprolactone (PCL) with improved mechanical properties for liver tissue regeneration by using a multi-head tissue building printing system. A co-cultured 3D microenvironment of primary rat hepatocytes (HCs), human umbilical vein endothelial cells (HUVECs), and human lung fibroblasts (HLFs) were successfully established and maintained to study liver cells proliferation. The results of this work suggested that the employed co-cultured microenvironment promoted heterotypic cellular interaction within a 3D construct. Similarly, Skardal et al. [77] utilized a 3D bioprinting platform to fabricate liver tissue, which has high potential for influencing how future drug and toxicology screening and personalized medicine approaches are performed. Measurable levels of both albumin and urea as well as common soluble biomarkers for liver were tested, and these remained relatively consistent throughout the culture period. Moreover, this group developed a 3D liver tissue model containing primary human hepatocytes and liver stellate cells supported by bioinks, and they tested the functional indicators. Specifically, these constructs were maintained in culture for six days, and liver functionality was examined by exposing the constructs to a hepatic toxicant, acetaminophen (APAP, 100 μM), and measuring the levels of albumin, urea, α-GST (alpha Glutathione S-Transferase), and lactic acid dehydrogenase (LDH) in the media over time. An analysis of both urea and albumin levels showed a significant decrease until day 15 for the acetaminophen-treated conditions. In addition, the levels of α-GST, a detoxification protein, increased at day nine, and the levels of lactic acid dehydrogenase (LDH), a marker of liver damage, also peaked due to printing-related stress but decrease to nominal levels by day six. Finally, histological staining presented a greater cellularity in untreated constructs, while drug-treated conditions showed a loss of cellularity. In the future, these models could be used for drug screening, disease modeling, and precision medicine applications [78]. An interesting decellularized extracellular matrix (dECM) bioink derived from a native liver was demonstrated by Lee et al. [79]. The proposed bioink, in combination with the 3D bioprinting technology, could be a suitable biomechanical and biochemical microenvironment for liver tissue function. Specifically, the cell-printed mixtures consisted of dECM bioink seeded with human bone marrow-derived mesenchymal stem cells (BMSCs) and liver cancer cells (human hepatocellular carcinoma), as well as PCL polymer for 3D structural support, with control constructs prepared with a collagen bioink. The resulting cell-laden printed bioink was evaluated and compared with those in commercial collagen bioink. An analysis of liver-specific functions of these constructs by assessing albumin and urea levels presented that the dECM bioink enhanced liver cell functions. Moreover, the level expression of key transcription factor HNF4A (Hepatocyte nuclear factor 4 alpha) was particularly upregulated in the liver dECM group to more than twice the level seen for the collagen, and the level expression of transcriptional markers HNF1A and HNF3B (Hepatocyte nuclear factor 3-beta) was significantly higher in the liver dECM group.

A recent study by Kurreck et al. [80] utilized the extrusion bioprinting technique to print a 3D tissue model composed of bioinks and human bipotent hepatic progenitor cells (HepaRG) with established applications in virus biology. A short summary of recent outstanding bioprinting studies is presented in Table 2.

**Figure 7:**
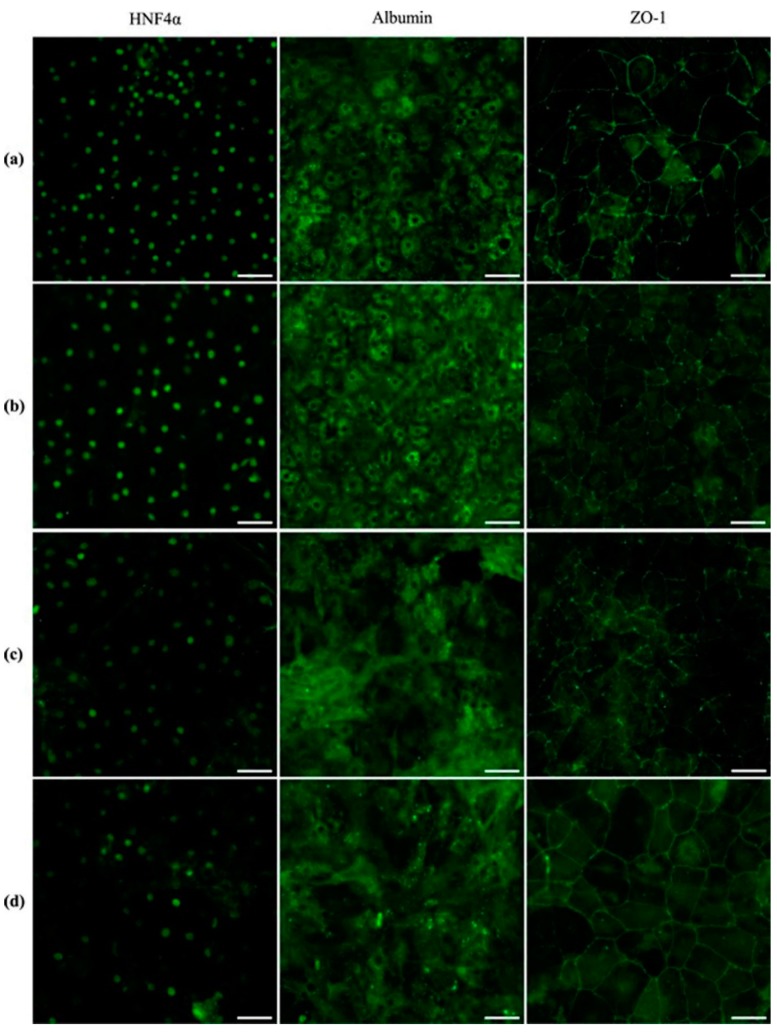
Fluorescence images of printed human-induced pluripotent stem cells (hiPSC)-derived hepatocytes showing hepatocyte marker expression in green: (**a**,**b**) human embryonic stem cells (hESC)-derived hepatocyte-like cells (HLCs) (RC-10): (**a**) Non-printed control; (**b**) printed results; (**c**,**d**) hiPSC-derived HLCs (RCi-22); (**c**) non-printed control; (**d**) printed results (scale bars 50 μm) [81].

An alternative approach to liver tissue fabrication is the use of stem cells. Concerning the hepatic differentiation of induced pluripotent stem cells (iPSCs) to liver-specific cell lines. The first successful work on bioprinting a mini-liver from both human-induced pluripotent stem cells (hiPSCs) and human embryonic stem cells (hESCs), which have matured to be hepatocyte-like cells, was reported by Faulkner-Jones et al. using a valve-based bioprinting system which did not adversely affect cell viability (~84%) [83]. The group built a 3D alginate matrix, and the analysis was carried out after 21 days of differentiation protocol, revealing peak albumin secretion that meant the construct was hepatic in nature [81], as shown in Figure 7 [84]. Recently, Choi et al. [85] used a nozzle 3D bioprinter to fabricate a liver-mimicking architecture using primary hepatocytes, and they demonstrated the benefits of co-cultured primary hepatocytes and mesenchymal stem cells (MSCs). This research indicated that the expression of hepatic genes and proteins was higher for up to seven days in the 3D hepatic architecture, and that the primary hepatocyte cell morphology was stable.

Most 3D-bioprinted tissues demonstrate liver-specific functions in addition to injury response. Several companies and research groups have created living constructs that mimic native liver structures and functions [86,87,88,89].

**Figure 8:**
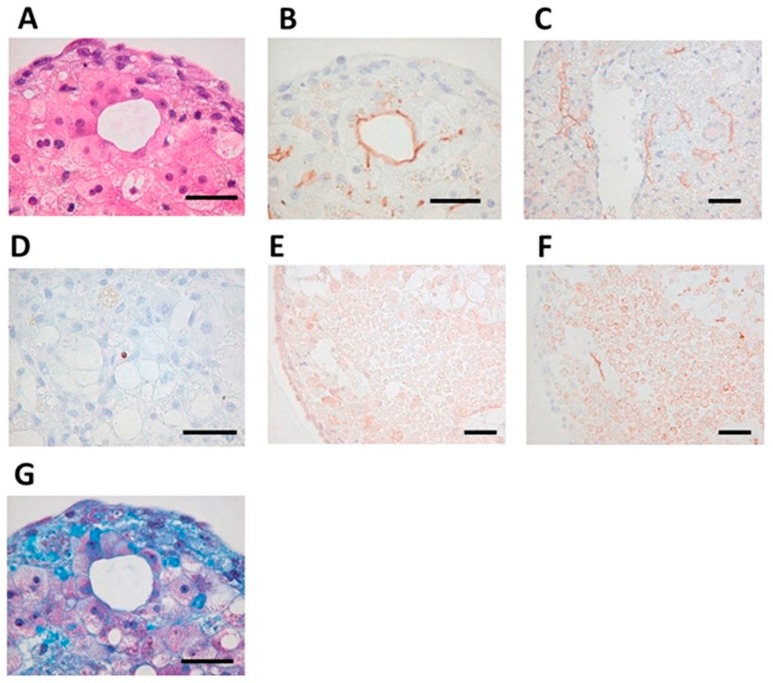
Self-organization in bio-printed human liver tissues. (**A**) Hematoxylin and eosin stain (HE) staining shows structure of bio-printed liver tissue on day 50. (**B**) Immunostaining with the MRP2 antibody detected bile acid transporters (day 50). (**C**) Immunostaining with, cluster of differentiation 31 (CD31) antibody detected blood vessel-like and sinusoid-like structures (day 14). (**D**) Terminal deoxynucleotidyl transferase dUTP nick end labeling (TUNEL) staining detected little apoptosis (day 60). (**E**) Immunostaining with the OAT2/8 antibody detected drug uptake transporters (day 44). (**F**) Immunostaining with MRP2 antibody showed tissue distribution (day 44). (**G**) Masson’s trichrome staining shows collagen accumulation (day 50). Black bars represent 50 μm [82].

There is an acute demand for livers, and the fabrication of liver tissue or liver will definitely alleviate this problem. Liver tissue and organoids can also be used in other assays such as drug testing and liver disease studies. As with mature hepatocytes, hepatocyte-like cells obtained from stem cells tend to quickly functionally deteriorate under in vitro conditions. The liver structure is complex with a modular microenvironment; thus, it is difficult to model native liver tissue [87]. Recently, Kizawa et al. [82] printed a liver tissue by the spheroid assembly of primary hepatocytes (1 × 104 cells/mL) that maintained functionality up to 60 days by using a scaffold-free 3D bioprinting technology from Cyfuse Biomedical (NA1002, Cyfuse Biomedical), as demonstrated in Figure 8. The human 3D bioprinted liver construct also maintained the expression of many drug transporter proteins and metabolic enzymes for many weeks.

Tissue engineers have continued to improve the quality of their human liver creations. The creation of living mini-organs is a relatively new area of science with the potential to replace animal models that are not always accurate. Organoid systems are the recently developed 3D bioengineered platforms for studying assays such as drug toxicity testing and metabolic diseases. Organoids are cell-derived in vitro 3D organ models that allow for the study of biological processes and also have important effects for clinical use in an environment that mimics endogenous cell organization and organ structures. These models overcome the major constraints of 2D tissue models and provide prolonged cell viability and functionality [90]. These in vitro culture systems contain a self-renewing stem cell population which differentiates into multiple, organ-specific cell types that exhibit a spatial organization similar to the corresponding organ and are capable of recapitulating some functions of that organ, thus providing a highly physiologically relevant system.

**Figure 9:**
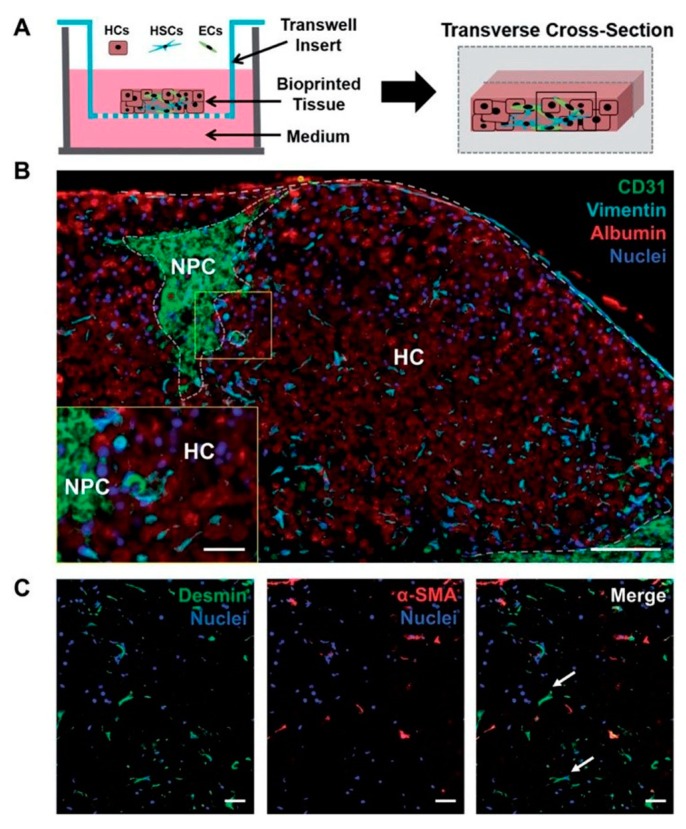
3D bioprinted tissue exhibits a compartmentalized architecture and maintains hepatic stellate cells in a quiescent-like phenotype. (**A**) Illustration of a transverse cross-section of bioprinted tissue on a transwell insert comprising hepatocytes (HCs) and compartmentalized endothelial cells (ECs) and hepatic stellate cells (HSCs). (**B**) The organization of non-parenchymal cells (NPCs) is depicted with CD31 and vimentin staining to mark ECs and HSCs, respectively. Albumin is used to denote the hepatocellular compartment (HC). Scale bar = 100 μm, inset scale bar = 25 μm. (**C**) HSC activation status was examined using desmin (generic marker) and Alpha-smooth muscle actin (α-SMA) (activation marker). Quiescent HSCs are denoted with white arrows. Scale bar = 50 μm [95].

Organoids have been formed via several different methods, e.g., spinner flask cultures [91], utilizing rotating cultures [92], stationary cultures in hanging drops with well-known 96- or 384-well plates [93], and cell growth on non-adherent surfaces [94]. The utilization of engineering tools such as biomaterial scaffolds, microfluidics and bioprinting has enabled greater control over the cellular environment, which has increased the accurate prediction of clinically relevant outcomes and the longevity of liver functions in vitro. For example, Norona et al. [95] fabricated a 3D bioprinted liver tissue housed in a 24-well Transwell (Corning Inc, Corning, NY, USA) that can recapitulate drug-, chemical-, and Transforming growth factor β1 (TGF-β1)-induced fibrogenesis at the cellular, molecular, and histological levels, as demonstrated in Figure 9. Taking into consideration the above characteristics, these bioprinted in vitro tissue models of human liver demonstrate the utility of novel 3D bioprinted tissues to further evaluate compound-induced liver fibrosis in a more defined and systematic way.

### 3.3. Liver-on-Chip Platforms

In contrast to static models, perfusion systems or cell microfluidic platforms can allow for the automated control over several conditions such as culture medium, pH, temperature, fluid pressures, cell shear stress, nutrient supply, and waste removal. Microfluidic systems have been implemented in engineering liver tissues [96]. Significant applications of microfluidics in tissue engineering technology include cell culture and making gradient biomaterials [97]. For these reasons, microfluidic cell platforms are preferable for mimicking the native and dynamic cellular environment compared to static cell culture systems [98]. Moreover, these systems remain precise long term and could provide information on tissue responses to various conditions over time scales that are clinically relevant [99].

The microarchitecture of the liver is crucial to liver function [100]. Hepatocytes interact with mesenchymal cells, stellate cells, Küpffer cells, macrophages, and lymphocytes [101]. A main feature of the liver is the perfusion of fluid. When compared to a conventional cell culture, liver function can be enhanced in a microfluidic chip [102].

Furthermore, some diseases or injury states have also been supported inside a microfluidic chamber for pharmaceutical testing [103,104]. Recently, polydimethylsiloxane (PDMS)-based microfluidic devices have been made obtainable by using multiple chambers to mimic the sinusoidal architecture of the liver. For example, Kang et al. [105] used their system to analyze the viral replication for hepatotropic hepatitis B virus. Moreover, they demonstrated that primary rat hepatocytes maintained normal morphology and produced urea for 30 days when they were cultivated on one side of a transwell membrane, while immortalized bovine aortic endothelial cells were cultivated on the other side of the membrane that was subjected to dual-channel microfluidic perfusion. Another group [106] developed a system to model alcohol injury. Their liver injury-on-chip system was made by two chambers for seeding of hepatocytes and stellate cells, as well as three more chambers for miniature aptamer-modified electrodes to monitor liver cell signaling. This system makes it possible to monitor the paracrine crosstalk between co-cultured cell types communicating via the same signaling.

Additionally, the advantages of perfusion on the functions of liver co-cultures is that perfusion can drive the cells to gradients of oxygen, nutrients, and hormones, which have been shown to lead to liver parenchyma or differential functions in hepatocytes across the length of the sinusoid [107]. Allen et al. [108] fabricated a perfusion bioreactor platform with oxygen gradients that was used to induce an in vivo-like zonal pattern of CYP450s and acetaminophen toxicity in rat hepatocyte cultures. This bioreactor system could provide useful information about the maintenance of liver zonation in order to get deeper insight into the mechanism of metabolism and toxicity.

In contrast to an oxygen gradient, McCarty et al. [109] demonstrated a gradient of exogenous hormone (insulin and glucagon) onto a rat hepatocyte monolayer using a microfluidic device. Utilizing this advanced control system, they demonstrated the in vitro creation of hepatocyte carbohydrate, nitrogen, alcohol degradation, and drug conjugation metabolic zonation. This useful type of system could be essential for the development of in vitro liver disease models.

**Figure 10:**
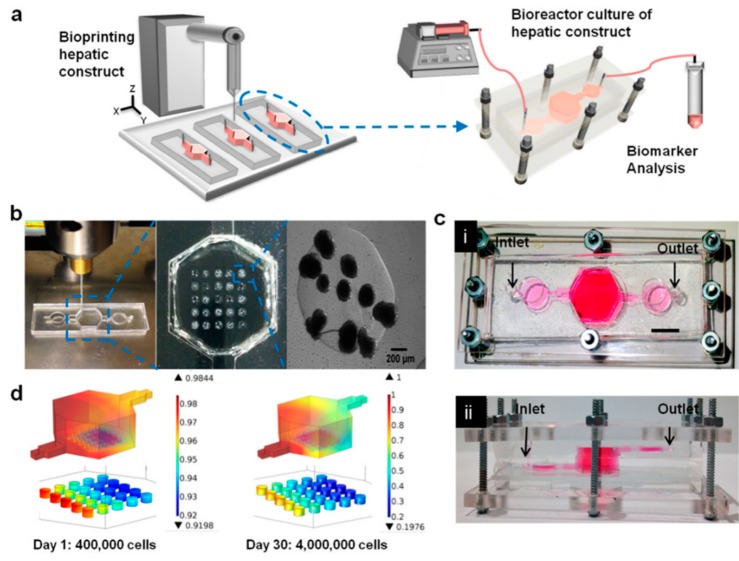
(**a**) Schematic of the hepatic bioreactor culture platform integrated with a bioprinter and biomarker analysis module. (**b**) Bioprinting photocrosslinkable Gelatin-methacryloyl (GelMA) hydrogel-based hepatic construct within the bioreactor as a dot array. (**c**) Top-view (i) and side-view (ii) of the assembled bioreactor with the inlet and outlet fluidic ports as indicated. Scale bar = 1 mm. (**d**) Oxygen concentration gradient in the bioreactor, considering the oxygen uptake of, case A: 400,000 hepatocytes on day one (16,000 cells per dot), and case B: 4000,000 hepatocytes on day 30 (160,000 cells per dot) [110].

Only a few reports have been published which combine direct printing techniques with on-chip technologies for the fabrication of organs on chips. Direct printing into a microfluidic chamber to build a liver-on-a-chip platform was also demonstrated by Bhise et al. [110]. Droplets of HepG2 spheroid- Gelatin-methacryloyl (GelMA) mixture were printed on a glass slide within the cell culture chamber of a bioreactor, followed by immediate UV cross linking. The engineered hepatic construct remained functional during the 30-day culture period and showed a drug response similar to published data (Figure 10).

Another printing technique utilizing micro valves integrated with microfluidic chips was studied by Chang et al. [111] in order to fabricate reproducible three-dimensional cell-encapsulated alginate-based, tissue-engineered constructs in chambers for drug screening platforms in planetary environments.

Liver platforms are being integrated with different cell lines for liver tissue fabrication. It has been researched that perfused hepatocyte-endothelial co cultures show a greater rate of production of drug metabolites relative to static controls [112]. An interesting in vitro hepatic model was demonstrated by Khetani et al. [60] for drug screening and modeling liver diseases using engineered micropatterned co-cultures of induced pluripotent stem cell-derived human hepatocyte-like cells (iHeps) and 3T3-J2 murine embryonic fibroblasts with a Matrigel. This in vitro model of human liver was maintained for several weeks in culture. Moreover, Cho et al. [113] developed a controlling co-cultured microenvironment to study the heterotypic cell interactions of hepatocytes on a patterned fibroblast layer using microfabricated PDMS stencils. The liver-specific functions of the hepatocytes including intracellular albumin staining and E-cadherin expression were increased as a result of enhanced heterotypic contact in the co culture system. In other similar research, primary human hepatocytes along with human endothelial (EA.hy926), immune (U937) and stellate (LX-2) cells were co-cultured in a microfluidic device. This study described a relevant liver model which was maintained for weeks in order to investigate liver studies and the microfluidic integration technology with other organs [114]. Other approaches to create artificial, three-dimensional hepatic tissue constructs and the regeneration of injured livers reported the co culture systems of hepatic stellate cells (HSCs) [115] and both HSCs and ECs [116]. Several liver platforms have already been fabricated with the aim of the reliable replication of liver physiology and metabolism to benefit the pharmaceutical industry in drug discovery and development. The performance of current liver platforms needs to be improved to further mimic the physiology and function of liver in the body. Future advances in this area could emerge from the combinatory use of existing technologies to move toward a liver model with a more complete functionality.

## 4. Scaffolds Fabrication Methods

Scaffolds are 3D artificial biostructures which are used in tissue engineering as well-defined matrices for cell adhesion and proliferation. A high porous architecture and a controllable porous size are key parameters for accommodating different types of cells, whereas porosity has a crucial role in attachment and migration of transplanted cells. Depending on the fabrication method and the raw material, the porous size varies between 100 and 500 μm in order to be suitable for applications such as bone regeneration [117], cardiac tissues [118] and cells proliferation [119].

Its biocompatibility, mechanical properties, and chemical properties make the material suitable for medical applications and cell culture. Towards the fabrication of 3D scaffolds, several approaches have been used, such as two-photon polymerization, selective laser sintering, and 3D printing techniques (inkjet and extrusion printing).

### 4.1. Laser-Based Methods

The main purpose for the fabrication of 3D structures that are aimed to be used as a matrix for the selective placement and growth of cells is the printing of biocompatible polymers for the creation of a 3D shape. Two photon polymerization, a widely used method for developing 3D materials suitable for cell growth and proliferation, is based on the irradiation of a monomer with a laser beam to trigger a cross-linking process by two photon absorption in selected depths [120]. As the desired structure forms by the selective polymerization offered by the laser beam, the non-polymerized monomer is subsequently removed by extensive washing procedures.

**Figure 11:**
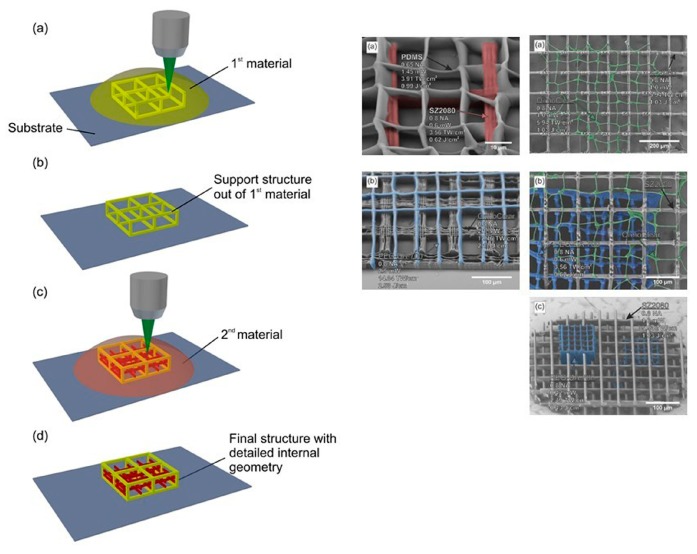
Direct writing laser procedure (**left**). Final structures of fabricated scaffolds consist of two different polymeric materials (**right**) [121].

The use of lasers for the creation of biopolymer scaffolds enables the easy tuning of the porosity of the final 3D structure by the alteration of the irradiation conditions, as explained by Rekštyte et al. [121]. In the reported study, 3D polymeric porous scaffolds with size porosity of micrometers were obtained with the use of four different combinations of materials and a large variety of fabrication parameters (Figure 11).

**Figure 12:**
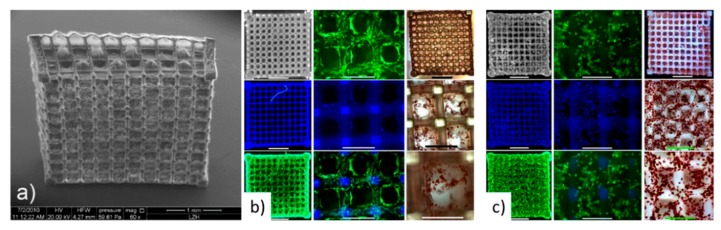
(**a**) SEM image of fabricated gelatin scaffold. (**b**,**c**) Fluorescence microscopy pictures for the 2 photon polymerization scaffold after seven days and 22 days [122].

In addition, Ovsianikov et al. at 2011 [122] created gelatin-based scaffolds with methacrylamide groups for the development of adipose tissue and transplants for plastic surgeries. The results verified the stability of the material and their ability to support ASC adhesion and proliferation from seven to twenty-two days as shown in Figure 12.

3D hydrogel scaffolds created by two-photon polymerization (2PP) for the support of Henrietta Lacks (HELA) cells’ culture for tissue engineering applications were also reported by Y.C Zheng et al. [123]. The starting material consisted of an aqueous solution of 3,6-bis[2-(1-methyl-pyridinium)vinyl]-9-pentyl-carbazole diiodide (BMVPC), cucurbit [7] uril (CB7), and polyethylene glycol diacrylate (PEGDA) was used as a monomer for 2PP.

**Figure 13:**
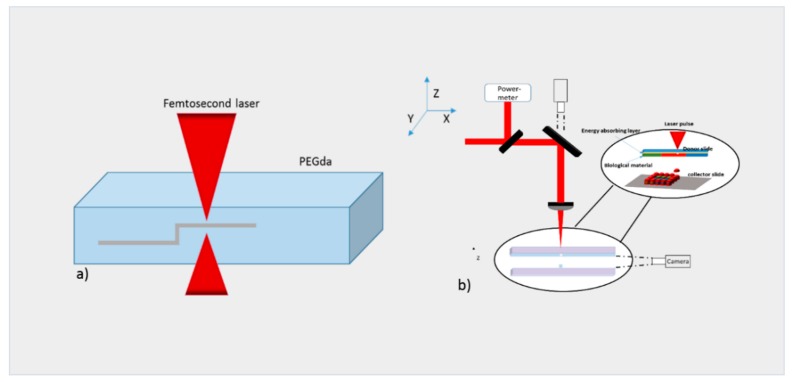
(**a**) Schematic representation of two photon polymerization process of an acrylated poly(ethyleneglycol) (PEG). (**b**) Schematic representation of LIFT technique for the printing of cells on the fabricated scaffold [120].

Another advantage that laser-based techniques offer is the use of lasers for the creation of the 3D matrix and the selective deposition of cells with high precision. Ovsianikov et al. [120] presented this approach by utilizing lasers to polymerize an acrylated poly(ethyleneglycol) (PEG) monomer for the fabrication of a cell scaffold and the direct laser printing of two different types of cells on the fabricated scaffold (Figure 13).

**Figure 14:**
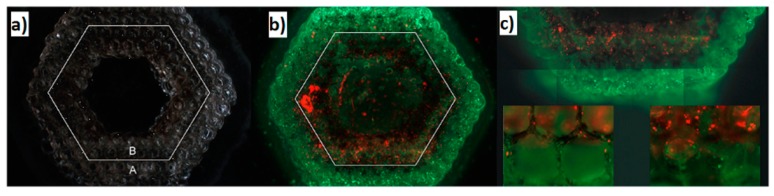
(**a**) SEM images of 2PP fabricated scaffolds. (**b**,**c**) Fluorescence microscopy images after the deposition of two cell lines in the same scaffold [120].

The final structure of this study had a hexagonal shape with six layers of cylinders along the diameter of the shape (Figure 14). Vascular smooth muscle cells (VSMCs) were laser printed at the outer perimeter of the scaffold, while EC cells were deposited at the inner perimeter (Figure 14).

**Figure 15:**
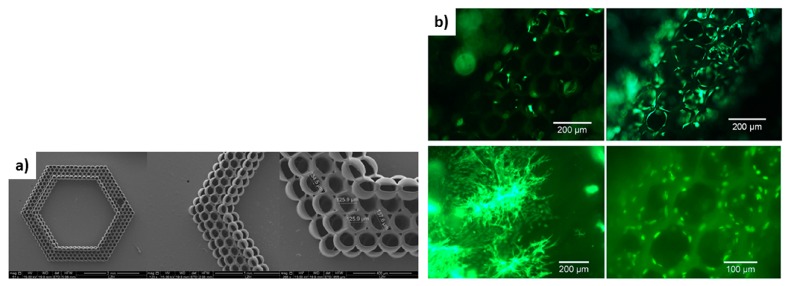
(**a**) Fabricated fibrin gel scaffold with hexagonal shape. (**b**) Cells cultured for seven days [124].

The combination of the laser-based 2PP technique with the micromolding technique resulted in the accelerated duration of the fabricated scaffolds, according to A. Koroleva et al. [124] (Figure 15). In this structure, human pulmonary microvascular endothelial cells (HPMEC) were cultured for seven days and migrated into the fabricated fibrin gel scaffold (Figure 15).

Another laser-based technique used for the fabrication of cell scaffolds is called selective laser sintering [125]. This is a layer-by-layer approach in which a laser beam is used to selectively sinter particles of a polymeric material in order to create layers with specific geometric characteristics [126].

### 4.2. Inkjet Printing

Inkjet printing is one of the most popular 3D printing techniques for the fabrication of structures of a great variety of materials. This technique enables the printing of picoliter droplets according to a software design in order to create 2D or 3D structures, and it can be either a continuous or a drop-on-demand (DOD) printing approach. The DOD inkjet printing technique has been widely used to create arrays of small liquid biodroplets [127,128]. The mechanism based on a thermal approach or a piezoelectric approach are the two main printing mechanisms with a DOD inkjet printer. The thermal inkjet printer contains of a thermal actuator which heats up the printing head, consequently generating a bubble of gas which, upon expansion, ejects a droplet of liquid to a receiver substrate [129]. On the other hand, the piezoelectric printers consist of a piezoelectric actuator which surrounds the ink chamber. An increase of the voltage across of the piezoelectric actuator initiates the formation of droplets during the flow of the ink [130].

**Figure 16:**
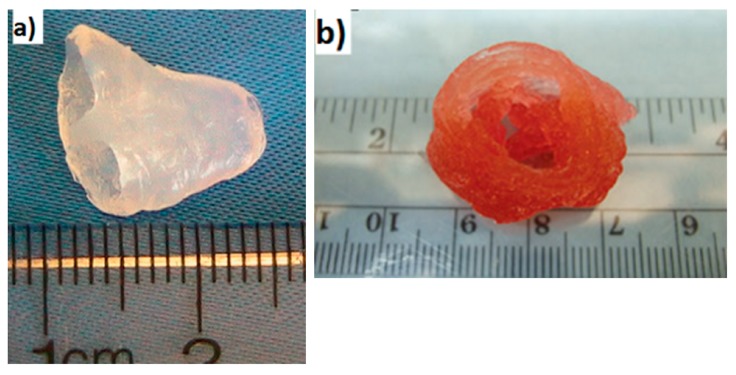
(**a**): Cardiac tissue by Xu et al. [132]. (**b**): Printed valve network by Duan et al. [54].

Ink jet printing techniques have been frequently used for the control of cells growth in a matrix by the printing of protein solutions [131], the printing of cells [131], or the printing of the 3D scaffold. In the field of inkjet printing of scaffolds, impressive results were presented by Xu et al. [132] with the printing of a functional 3D scaffold for cardiac tissue application (Figure 16a). Also, Duan et al. who printed valve network (Figure 16b) [54].

Moreover, there have been studies that utilize the inkjet printing technique for the creation of a 3D polymer matrix and the precise deposition of cells in a twostep procedure [133].

Even though the inkjet printing technique is an established technique for the printing of materials that can be used as 3D scaffolds for cell cultures, such as biodegradable polymers [134,135,136] and natural polymers [133,137], most of the reported 3D liver cell cultures by the use of scaffolds are created by more verified techniques in the industry such as microextrusion printing. Microextrusion printing is an additive manufacturing method for creating 3D micro-structures in a layer-by-layer manner. This is enabled by the continuous microprinting of polymeric materials for the creation of individual layers. These types of printers consist of a piston, upon which extraction deposits the biomaterial through a micro-needle. To our knowledge, the only study in which a piezoelectric inkjet printer was used for creating scaffolds for liver cultures was presented by Arai et al. [73]. The novelty of that work was the architecture of the final structure—a sandwich shape of two galactosylated alginate (GA)-gel sheets on the top and bottom and hepatocytes cells in-between the two layers. This design provided the opportunity to regulate the polarity of the hepatocytes.

## 5. Scaffolds for Liver Tissue Engineering

As mentioned previously, the liver is one of the most important and largest organs in human body, and it plays a significant role in metabolic functions. Many groups have studied the generation of 3D liver structures for potential liver regeneration applications. The use of 3D polymeric structures for generating cell cultures and liver models has facilitated the overcoming of the limitations of 2D cell culture models such as the uncontrollable cells’ polarity and non-directed cells’ attachment. A wide range of biocompatible polymers has been used as starting materials for building stable matrices for the growth and proliferation of cancer and primary hepatic cell lines. Lewis et al. [138] investigated the creation of a 3D porous gelatin scaffold using a pneumatic extrusion piston-driven EnvisionTEC (GmbH) 3D-Bioplotter.

Six different single layers with different porous sizes were precisely placed in such a way to create two variable geometries (differing in the strength of the connection between the layers), towards the final 3D scaffold. The scaffolds were used as matrix for the cell culture of the differentiated hepatocyte-derived carcinoma cell line (Huh7). After, the fabrication of the optimum scaffold geometry, the viability and the functionality of the seeded Huh7 cells were studied for seven days. Towards this goal, a comparison was made between the two geometries of the 3D scaffolds and the 2D models. The results revealed almost the same viability of the 2D and 3D cell cultures; however, the 3D structures enabled an increase in the hepatic functions of the cells, mainly due to the strong connection between the pores of the structure. These lateral architectural 3D models have been proven suitable to use for studying the specific functions of hepatocytes such as albumin secretion, CYP activity, and bile transport, because they provide an appropriate environment for well-defined cells [138]. Furthermore, a micro-extrusion bioprinter (INKREDIBLE+) was used by Hiller et al. to create a 3D structure by printing a mixed ink consisting of: hydrogels (alginate, gelatin), human extracellular matrix (hECM) and human HepaRG liver cells. The selected cell line was used in thus metabolic study due to its morphology and metabolic characteristics. The aim of this study was to test the metabolic activity and the viability of the cells for structures with variable concentration of hECM. The hECM substance changes the mechanical characteristics of the 3D microstructures. It consists of collagen type I, which improves the properties of the scaffold but, in high concentrations, has a negative effect on cell functionality [80]. Another group (Kim et al.) used alginate and isolated mouse primary hepatocytes to create a 3D bio-printed structure. The process combined the use of a micro-syringe with a three dimensional motion stage to create the final 3D structure in a layer-by-layer manner. The final shape of the structure was adjusted by scanning parameters such as velocity and pressure. The main goal of this study was to create hepatocyte cell culture networks which demonstrated a high viability of the cells after 14 days with good hepatic functionality [75]. The same year, Kang et al. [139] used mouse-induced hepatocyte-like cells (miHeps) by pluripotent stem cells (PSC) for the development of a 3D structure made by miHeps and aginate using extrusion printing. Five layers of cells and alginate printing solution built the final 3D cell culture which was placed in a mouse in vivo. The implant was examined 14 and 28 days after the surgery, presenting results that indicated that the in vivo transplanted scaffold was more functional than the in vitro model. Lee et al. [76] created 3D scaffolds with improved mechanical properties for a 3D hepatocytes cell culture environment. In this study, the scaffold was made by polycaprolactone (PCL) as a starting material by using a homemade printer with multiple deposition heads-multihead tissue/organ building (MtoBS system). The layer-by-layer printed structure consisted of PCL and hydrogel layers with a mixture of collagen and three different cell lines—HCs, HUVECs, and HLFs. The final structures had the ability to support multiple functional cell lines which could maintain their hepatic functions for 10 days. Jeon et al. fabricated 3D alginate scaffolds with cancer hepatic cells (HepG2) using a micro extrusion printer and tested the proliferation and the viability of hepatocytes on the 3D structure for three weeks. The histology and immunohistochemistry of the final cultures were investigated along with their ability to support operational cells [140]. Gong et al. designed and fabricated well defined 3D chitosan–gelatin (C/G) scaffolds which consisted of polymeric channels and pores using both an indirect method called the solid freeform fabrication (SFF) and freeze drying methods. Two thermoplastic materials were used for forming the mold of the scaffold. Chitosan and gelatin were used as matrix materials, and the final structure was initiated by the freeze-drying process, which also led to the creation of micro pores. The tuning of the freeze-drying parameters resulted in the optimum shape and morphology of the scaffold. The functionality of the developed scaffold structure was tested for the culturing of HepG2 cell line [141].

## 6. Conclusions

In summary, 3D bioprinting technology enables the fabrication of biomimetic tissues and implants with the use of biomaterials, growth factors, and living cells, which can either be printed in a specific pattern for the development of the final tissue structure or, in many cases, can be printed on an already existing 3D matrix (scaffold). Furthermore, in the field of tissue engineering, an bioartificial liver is considered one of the most promising tools as a therapeutic method for severe liver diseases and, in the field of regenerative medicine, for drug testing. The most commonly used direct writing techniques for the printing of cells are laser-based techniques, inkjet printing, and microextrusion printing. Those techniques are mainly chosen because they can easily adapt to the cultivation environment, can create high-resolution cell structures, and, in many cases, can also be used to create 3D scaffolds for the cell growth. In the field of liver tissue engineering, a lot of work has been done with the use of the above-mentioned techniques towards; however, greater effort is required to solve problems encountered in the inability to replicate the actual 3D living liver tissue environment. Finally, the combination of a 3D bioprinting technique with a microfluidic control can be a promising method for controlled drug delivery systems and for future regenerative medicine.

## Figures and Tables

**Table 1:** A brief review of common bioprinting techniques.

	Laser Assisted Bioprinting	Inkjet	Extrusion
Advantages	High resolution, deposition of biomaterials in solid or liquid phase, and nozzle free and non-contact printing.	Ability to print low viscosity biomaterials, fast fabrication speed, low cost, high resolution, multi-material printing, Simple operation.	Simple, capable of printing various biomaterials, ability to print high cell densities, multi-material printing, and ability to control ejection speed.
Drawbacks	High cost, thermal damage due to nanosecond/femtosecond laser irritation, metallic residuals possible damage of tissue from use of laser lights, slow printing speed, and difficulty in handling heterogenous cells.	Inherent inability to provide a continuous flow, poor functionality for vertical structures, low cell densities, clogging of nozzle, imposing thermal or acoustic stress to cells, and limited variety of bioink.	Only applicable for viscous liquids, gelation and solidification, and limited material selection (shear thinning ability required).
Speed	Medium	Fast	Slow
Cell viability	<85%	~80%	>90%
Resolution	10 μm	50 μm	100 μm
Cell density	Medium	Low	High
Viscosity	1–300 mPa s	<10 mPa s	30–6 × 10^7^ mPa s
Scalability	Low	Low	Low–Medium
Structural integrity	Low	Low	High
Cost	High	Low	Low–Medium

**Table 2:** A short summary of outstanding recent liver bioprinting studies.

Printing Method	Cell Type/Bioink	Achievements	Reference
Extrusion bioprinting	Hepatocytes Gelatin	The laminated hepatocytes remained viable and performed biological functions for more than 2 months	[68]
Extrusion-based bioprinting	Primary human hepatocytes, hepatic stellates, HUVEC cells, and non-parenchymal cells/NovoGelR 2.0 hydrogel (concentration not mentioned)	Viable up to 28 days (% not mentioned) Inkjet bioprinting Galactosylated alginate (12 mg/mL) Primary mouse hepatocytes (isolated from the liver tissue of male 6–8-weeks-old ICR 12 mice) Data not available >85% after 2 Days test of hepatotoxicity of trovafloxacin and Levofloxacin	[71]
Inkjet bioprinting	Primary mouse hepatocytes (isolated from the liver tissue of male 6-to-8-week-old ICR 12 mice)/Galactosylated alginate (12 mg/mL)	>85% after 2 days	[73]
Inkjet bioprinting	HUVEC	Multilayered organ tissue model test of hepatotoxicity of troglitazone (Rezulin)	[74]
Extrusion-based bioprinting	Primary mouse hepatocytes (isolated from the livers of 6–8 weeks old mice)/Alginate (3% *w*/*v*)	Viable up to 14 days (% not mentioned)	[75]
Extrusion bioprinting	HepG2, BMMSCs/decellularized extracellular matrix (dECM)	Liver tissue model	[79]
Microvalve bioprinting	hiPSCs (human-induced pluripotent stem cell lines, RCi-22 and RCi-50); hESCs human embryonic stem cell lines, RC-6 and RC-10)/Alginate (1.5% *w*/*v*)	>55% after 1 day	[81]
Extrusion-based bioprinting	Primary hepatocytes	Viable up to 60 days (% not mentioned)	[82]
